# Whole-Genome sequencing and comparative genomics of *Mycobacterium* spp. from farmed Atlantic and coho salmon in Chile

**DOI:** 10.1007/s10482-021-01592-w

**Published:** 2021-05-30

**Authors:** Rudy Suarez, Karina Kusch, Claudio D. Miranda, Tianlu Li, Javier Campanini, Phani Rama Krishna Behra, Luis Aro, Alexis Martínez, Marcos Godoy, Daniel A. Medina

**Affiliations:** 1 Centro de Investigaciones Biológicas Aplicadas, Diego de Almagro Norte 1013, Puerto Montt, Chile; 2 Programa de Magíster en Acuicultura, Facultad de Ciencias del Mar, Universidad Católica del Norte, Larrondo 1281, Coquimbo, Chile; 3 Laboratorio de Patobiología Acuática, Departamento de Acuicultura, Universidad Católica del Norte, Larrondo 1281, Coquimbo, Chile; 4 Epigenetics and Immune Disease Group, Josep Carreras Leukaemia Research Institute, Badalona, Barcelona, Spain; 5 Facultad de Medicina y Ciencia, Universidad San Sebastián, Lago Panguipulli 1390, Puerto Montt, Chile; 6 Department of Cell and Molecular Biology, Biomedical Centre, Box 596, 751 24 Uppsala, Sweden; 7 Benchmark Genetics, Santa Rosa 560 oficina 25 B, Puerto Varas, Chile; 8 Cardonal S/N, AquaChile, Puerto Montt, Chile; 9 Programa Cooperativo Doctorado en Acuicultura, Universidad Católica del Norte, Larrondo 1281, Coquimbo, Chile; 10 Laboratorio de Biotecnología Aplicada, Facultad de Medicina Veterinaria, Universidad San Sebastián, Lago Panguipulli 1390, Puerto Montt, Chile

**Keywords:** Aquaculture, Comparative genomics, Emerging pathogens, *Mycobacterium*, Whole-genome sequencing, Chile

## Abstract

Several members of the *Mycobacterium* genus cause invasive infections in humans and animals. According to a recent phylogenetic analysis, some strains of *Mycobacterium salmoniphilum* (*Msal*), which are the main culprit in bacterial outbreaks in freshwater fish aquaculture, have been assigned to a separate branch containing *Mycobacterium franklinii (Mfra)*, another species that causes infections in humans. However, this genus is little studied in an aquaculture context. Here, we isolated four *Mycobacterium* spp. strains from freshwater cultures of Atlantic and coho salmon in Chile and performed whole-genome sequencing for deep genomic characterization. In addition, we described the gross pathology and histopathology of the outbreaks. Several bioinformatic analyses were performed using the genomes of these four *Mycobacterium* isolates in conjunction with those of *Msal* strains, four *Msal*-like strains, and one *Mfra* strains, plus 17 other publicly available *Mycobacterium* genomes. We found that three isolates are clustered into the *Msal* branch, whereas one isolate clustered with the *Mfra*/*Msal*-like strains. We further evaluated the presence of virulence and antimicrobial resistance genes and observed that the four isolates were closely related to the *Msal* and *Msal*-like taxa and carried several antimicrobial resistance and virulence genes that are similar to those of other pathogenic members of the *Mycobacterium* clade. Altogether, our characterization *Msal* and *Msal-like* presented here shed new light on the basis of mycobacteriosis provides quantitative evidence that *Mycobacterium* strains are a potential risk for aquaculture asetiological agents of emerging diseases, and highlight their biological scopes in the aquaculture industry.

**Supplementary information:**

The online version contains supplementary material available at (10.1007/s10482-021-01592-w).

## Introduction

Mycobacteriosis refers to infections caused by some members of the bacterial genus *Mycobacterium*. In fish, mycobacteriosis is characterized as a chronic progressive disease, with several external signs such as emaciation, inflammation of the skin, exophthalmia, ulceration, and open lesions (Austin and Austin 2016). Commonly, *Mycobacterium* are found in a wide range of environmental niches, particularly in aquatic environments, and only a small percentage of these species cause diseases in humans and animals. Fish mycobacteriosis may take several years to progress from an asymptomatic state to clinical illness and can affect more than 150 fish species, both farmed and wild (Decostere et al. 2004; Gauthier and Rhodes 2009; Heckert et al. 2001; Zanoni et al. 2008). For example, *Mycobacterium pseudoshottsii* has recently been identified as the etiological agent of outbreaks in three farmed fish species (*Dicentrarchus labrax*, *Sparus aurata*, and *Sciaenops ocellatus*), thus indicating that various farmed fish species are affected by *Mycobacterium* (Mugetti et al. 2020).

Currently, more than 170 distinct species have been identified within the *Mycobacterium* genus (Forbes 2017), and three of these, namely *M. marinum (Mma)*, *M. fortuitum (Mfor)*, and *M. chelonae (Mche)*, have been recognized as major causes of mycobacteriosis in fish (Gauthier and Rhodes 2009; Gcebe et al. 2018; Heckert et al. 2001; Rhodes et al. 2001; Whipps et al. 2008; Talaat et al. 1999). The typical gross pathology of mycobacteriosis in fish includes non-specific phenotypic changes, such as lethargy and anorexia, cutaneous ulcers, and decolouration (Bruno et al. 1998; Brocklebank et al. 2003; Luo et al. 2018). Several case studies have identified the presence of multiple granulomas in the skin and internal organs (Bruno et al. 1998; Brocklebank et al. 2003; Luo et al. 2018; Keller et al. 2018).

Recently, the list of species causing piscine mycobacteriosis has been expanded to include *Msal,* a taxonomically controversial specie. *Msal* was first classified in 1960 following its isolation from the kidneys of salmonid fishes and was described as an acid-fast bacillus (Ross 1960). However, owing to its biochemical similarities with *M. fortuitum* (Gordon and Mihm 1959) and the fact that two species could not be distinguished from each other, *Msal* was subsequently omitted as a separate *Mycobacterium* species (Skerman et al. 1989). Thereafter, following subsequent advances in molecular techniques, Whipps et al. (2007) have performed a phylogenetic analysis of the SSU rRNA genes of the strains isolated from salmonid fishes and confirmed that *Msal* is a monophyletic species, which is included within the *Mche*–*M. abscessus (Mabs)* complex (MCAC), which contains many clinically relevant human pathogens but *Msal* has not been implicated as a cause of disease in humans (Simmon et al. 2011).

Advances in molecular techniques allowed genomic analysis with single-nucleotide resolution and thus propelled the characterization and identification of *Msal*. Zerihun et al. (2011a) were the first to identify an *Msal* strain isolated from infected Norwegian salmon. By aligning the partial sequences of the 16S rRNA, *rpoB*, and *Hsp65* genes, the isolated strain was found to be 95–99% similar to *Msal* ATCC 13758. Later, Aro et al. (2014) utilized a similar strategy to identify the strain they isolated from an infected Chilean *Salmo salar,* and they showed that the 16S rRNA gene sequence obtained from *Msal* isolates shared 100% similarity with those from the *Msal* DQ866770 and *Msal* DQ866766 strains. In addition, these studies have found that the infected fish displayed significant macroscopic symptoms, such as external ulcers, poor body condition, pale gills, and erosion in the pectoral fin. Internal lesions, loss of adipose tissues, liver discolouration, and swelling of the internal organs have also been reported (Zerihun et al. 2011b, a; Aro et al. 2014). Nevertheless, both studies lacked of a genome-wide characterizations of the isolated strains. whole-genome.

The ability of *Msal* to infect mammals was first tested in vitro by Harriff et al. (2008). They observed that several *Msal* strains could infect and grow in both mouse and human macrophage cell lines. More recently, *Msal* infection has been reported in mice, in which an outbreak in an animal facility at Uppsala University was confirmed through deep DNA sequencing to have been caused by *Mycobacterium* species (Behra et al. 2019). The authors identified one as *Msal,* and the other three strains as *Msal*-like, which they proposed as new species of *Mycobacterium* after performing comparative genomics with 36 other MCAC members. Another recent study detected the presence of *Renibacterium salmoninarum* and *Mycobacterium spp*. in wild brown trout in Austria during summer. Owing to the importance of *R. salmoninarum* in aquaculture as an etiological agent of bacterial kidney diseases, the presence of both pathogens in wild fish could present a potentially serious infectious disease risk for the aquaculture industry (Delghandi et al. 2020).

The objective of this study was characterizing the macroscopic and microscopic traits of mycobacteriosis and performing whole-genome sequencing on the four *Mycobacterium* strains isolated from *S. salar* and *Oncorhynchus kisutch* salmon aquaculture facilities, presenting both kind of results, in silico and in vivo findings simultaneously that further describe the pathogenicity of mycobacteriosis. In addition, we compared the genomic and functional features of these strains with those found in several publicly available *Mycobacterium* genomes. In overall, this study gives novel insight regarding the identification and deep characterization of *Mycobacteryum* isolates using a combination of several macroscopic, microscopic, phenotypic, and genomic approaches.

## Materials and methods

### Ethical statement

Tissue sampling and fish manipulation was performed under the Guidelines for the Use of Fishes in Research (Nickum 2004) and the Care and Use of Fish in Research, Teaching and Testing (Canadian Council on Animal Care 2005).

### Strain isolation, cultivation, and characterization

Three mycobacterial strains (myc161, myc162 and myc182) were isolated from *S. salar* and one (myc151) was isolated from *O. kisutch,* both of which were obtained from freshwater farms located in the IX and X regions of Chile between 2015 and 2018 (Table 1). The bacterial isolates were recovered following the method described by Zerihun et al. (2011a, b). Briefly, bacteria were recovered from the livers of infected fishes in which grey-white nodules were present. Pieces of infected tissue were carefully disaggregated and suspended in sterile phosphate-buffered saline (PBS). Volumes of 1 mL of serial dilutions (ranging from 10^–5^ to 10^–7^ of the initial solution concentration) were plated on tryptic soy agar medium (TSA; Becton Dickinson, USA), and plates were incubated at 25 ºC for 5 to 10 days (Austin and Austin 2016). White creamy, brilliant, slightly convex colonies were subsequently transferred to modified Anacker and Ordal′s agar (MAOA) medium as previously reported (Zerihun et al. 2011b; Aro et al. 2014). Growth sensitivity to temperature was evaluated by culturing the isolates at 16, 25, and 37 °C for 5 days on solid TSA, MacConkey agar, brain and heart infusion agar (BHI), MAOA, blood agar (BA), and TSA supplemented with 5% NaCl. Biochemical characteristics of the strains were determined using the Analytical Profile Index (API) 20E® system (BioMérieux, France) following the manufacturer’s instructions.

**Table 1:** Descriptions of the four strains

Strain	Region	Recovered from	Year	Life stage	water	weight (g)	Gross pathology	associated diseases
myc161	IX	*S. salar*	2016	Smolt	River	3500	Yes	No
myc182	X	*S. salar*	2018	Fry	Watershed	80	No	No
myc162	X	*S. salar*	2016	Broodstock	River	3600	Yes	No
myc151	X	*O. kisutch*	2015	Fry	Lake	100	No	Flavobacteriosis

### Histopathologic analysis

Tissue samples from the gills, heart, liver, kidney, spleen, stomach, intestines, and pyloric caeca were collected from euthanised fish and fixed in 10% buffered formalin to prepare them for analysis. The fixed tissues were processed as previously described (Prophet 1992), and paraffin-embedded samples were sectioned into 3- to 4-μm blocks. The sections were mounted on glass slides and stained with haematoxylin and eosin according to previously described protocols (Fischer et al. 2008). Microscopic examination was carried out using a Leica DM2000 microscope (Leica, Germany) and visualized utilizing LAS software V3.3–2019 (Leica).

### DNA isolation and sequencing

Bacterial DNA extraction was performed using a modified protocol consisting of phenol chloroform and tissue homogenization using glass beads (Anahtar et al. 2016). To facilitate bacterial wall rupture, enzymatic digestion was performed with lysozymes at 37 °C for 1 h, followed by Proteinase K incubation at 55 °C overnight prior to bead-beating homogenization (Gill et al. 2016). DNA integrity was analysed using 1% agarose gel electrophoresis, and nucleic acid quantification was performed using a NanoDrop Lite (Thermo Scientific, USA). To confirm that the isolates belong to the *Mycobacterium* genus, a PCR assay was performed using the 27F and 1492R primers for the amplification of the 16S rRNA gene (Weisburg et al. 1991). PCR products were analysed using Sanger sequencing with an ABI PRISM 3500 XL sequencer (Applied Biosystems, USA), and the obtained sequences were used for BLAST analysis (Altschul et al. 1990). For whole-genome sequencing, 2 µg DNA was sequenced by the Novogene Corporation Inc. (Sacramento, USA) on an Illumina Novaseq 6000 platform using 2 × 150 paired-end. The raw data reads obtained from the Illumina platform have been deposited at the ENA-EMBL database under the accession number PRJEB38737 (Table 2).

**Table 2:** Summary of genome annotations, coding sequences, and accession number of whole-genome sequences produced in this study

Isolate	Reads	GC content (%)	Assembly size (bp)	Coverage	No. CDS	ENA Accesion n.º
myc161	14,727,378	64	4,582,212	964	4434	ERS4631745
myc182	15,638,477	63	4,391,176	1068	4277	ERS4631746
myc162	15,366,177	63	4,371,854	1054	4268	ERS4631747
myc151	17,400,957	64	3,993,798	1307	4080	ERS4631748

### Quality control, assembly, and genome annotation

Raw sequences generated in this study and those from a previous report (Behra et al. 2019) were pre-processed using Trimmomatic v0.39 to remove the leading and trailing bases with quality <28 and drops reads <120 bases long. Bases with average quality scores below 20 were trimmed (Bolger et al. 2014). The filtered reads were mapped against the *Msal* DSM43276 genome using SMALT aligner v0.7.6 available at the Sanger Institute (https://www.sanger.ac.uk/science/tools/smalt-0). SAMtools and BCFtools (http://www.htslib.org) were used to generate the consensus FASTA sequences (Li et al. 2009). The genomes were annotated using Prokka v1.12 (Seemann 2014), and the core- and pan-genomes were assessed using Roary v3.12.0 with a minimum percentage identity of 95% for blastp (Page et al. 2015). The core- and pan-genome were drawn using the script roary2svg.pl as described in the Roary pan-genome pipeline (https://sanger-pathogens.github.io/Roary/).

### Comparison of *Mycobacterium* genomes

Full-length 16S rRNA sequences from our four newly sequenced genomes and from 30 additional mycobacterial genomes retrieved from the NCBI database (Supplementary Table 1) were extracted using metaxa2 v2.2.1 (Bengtsson-Palme et al. 2015), and phylogenetic dendrograms were constructed using the Decipher package (Wright 2016) in R environment (R Core Team 2013). The full-length *rpoB* gene sequences obtained from the Prokka annotation were used for phylogenetic classification. The evolutionary distance at the species level was assessed using average nucleotide identity (ANI) analysis of the homologous genomic regions, and the 28 publicly available *Mycobacterium* complete genome sequences were compared using pyani module v0.2.10 (Pritchard et al. 2016). For this purpose, genomes were aligned using the MUMmer tool v3.23 (Kurtz et al. 2004).

### Functional annotation of orthologous genes

The sequences previously obtained from the Prokka analysis were annotated against NCBI’s cluster of orthologous groups (COG) database using the web tool WebMGA (Wu et al. 2011), and orthologous clustering was subsequently performed using OrthoVenn2 (Xu et al. 2019) (https://orthovenn2.bioinfotoolkits.net/). Unique elements were retrieved for orthologous classification from OrthoVenn2, and their sequences were used for protein identification in Blastp (Altschul et al. 1990). The virulence and antimicrobial resistance genes were identified using ABRIcate v0.9.8 (https://github.com/tseemann/abricate) by means of the NCBI Bacterial Antimicrobial Resistance Reference Gene Database (Feldgarden et al. 2019), Resfinder (Zankari et al. 2012), and the Virulence Factors of Pathogenic Bacteria database (VFDB) (Chen et al. 2016).

## Results

### Anatomopathological and microscopic findings

**Fig. 1:**
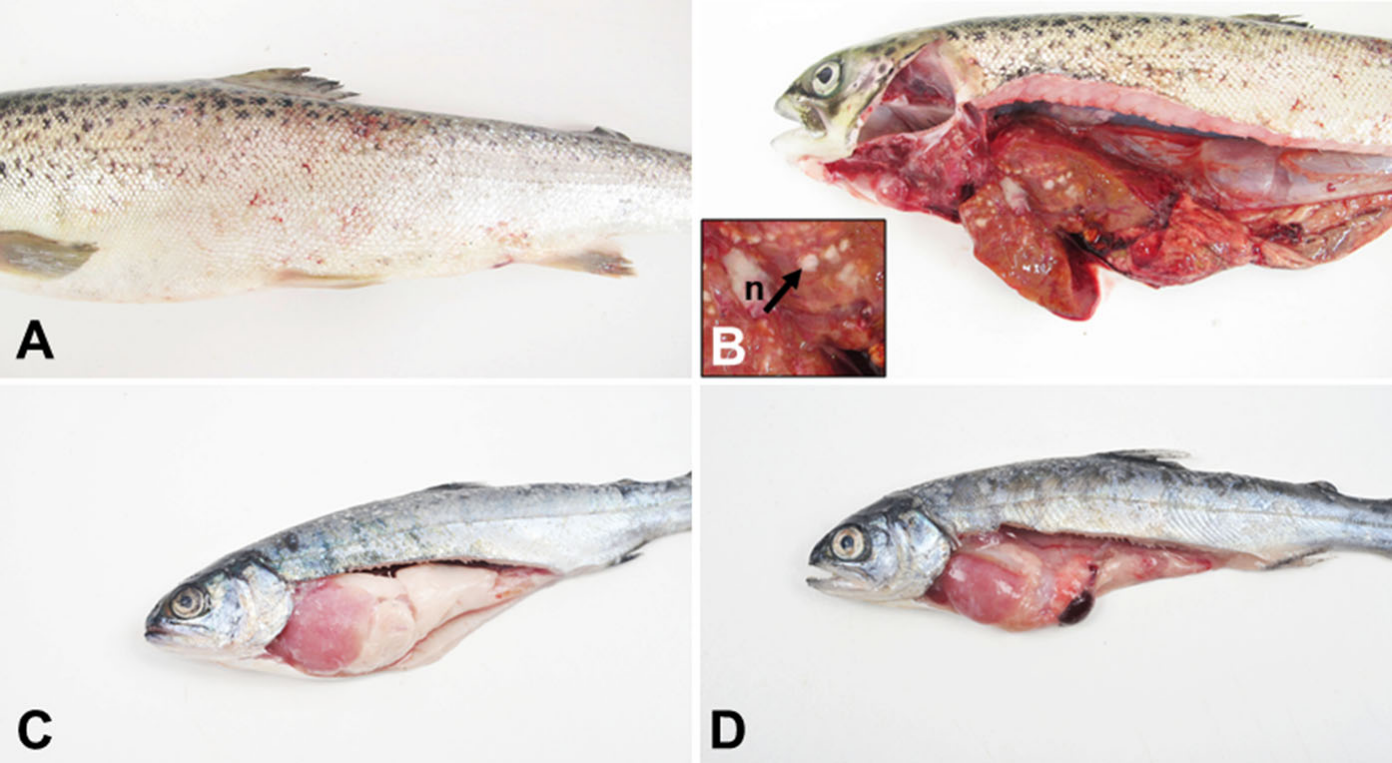
Macroscopic findings of mycobacteriosis. (**a**) Skins lesion on *S. salar*. (**b**) internal overview of S. salar. (**c** & **d**) *O. kisutch* internal overview

Three *Mycobacterium* strains were isolated from *S. salar* and *O. kisutch*, respectively, that were obtained from freshwater farms in the south of Chile. Macroscopically, we found raised scales in both salmonid species, as shown for *S. salar* (Fig. 1A). Internally, the fish presented hepatomegaly, splenomegaly, and posterior renomegaly. In the liver, the presence of pseudo-membranes with opaque colouration was recurrent. Histopathologically, the fish presented multiple foci of mononuclear cells in the liver and variable infiltration of mononuclear cells in the liver and spleen parenchyma. In both species, food was not detected in the digestive system of the diseased fish.

**Fig. 2:**
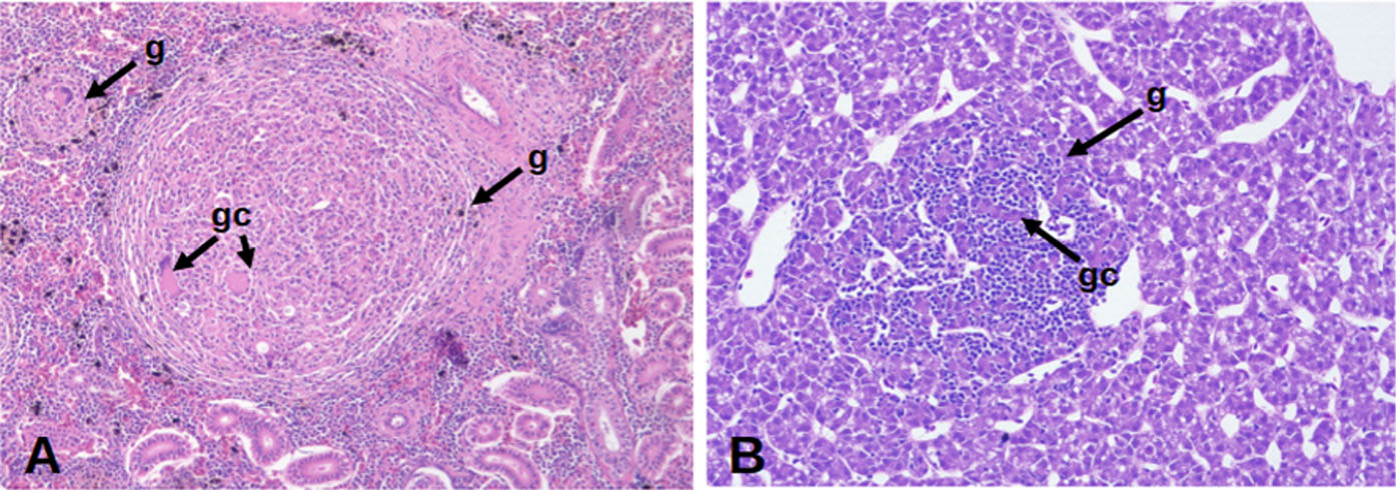
(**a**) Kidney and liver (**b**) H&E granulomas at 40 ×. Arrows indicate white nodules (n), granulomas (g), and giant cells (gc)

Specifically, the mycobacteriosis on *S. salar* was characterized by pale gills with multiple white nodules. Internally, we also observed the accumulation of visceral fat with signs of hepatomegaly, renomehalia, splenomegaly, and variably shaped white nodules in the kidney, liver, and spleen (Fig. 1B). Histologically, we observed multiple granulomas composed of mononuclear cells, surrounded by fibroblasts, giant cells, and multifocal granulomatous nephritis (Fig. 2a).

There were some stark differences with the mycobacteriosis in *O. kisutch*. Mainly, inner peritonitis was observed (Fig. 1c). Internally, the fish showed hepatomegaly, splenomegaly, posterior renomegaly, accumulation of visceral fat, ascites, and the presence of pseudo-membranes in the liver and spleen (Fig. 1c and d). Histopathologically, in the liver, multiple foci of mononuclear cells (Fig. 2b) and fibrin deposits in its capsule were detected, consistent with subacute to chronic multifocal hepatitis. In addition, the infiltration of mononuclear cells in the splenic and renal parenchyma was observed, which indicate diffused, subacute, and mild to moderate nephritis and splenitis. Also, cutaneous flavobacteriosis infection symptoms were observed in this species.

### Microbiological characterization

After 5 days of incubation in the MAOA medium, we obtained pure white, circular, smooth and well-defined colonies (Supplementary Fig. 1a). Ziehl–Neelsen staining results revealed that all strains were rod acid-fast bacteria (Supplementary Fig. 1b) similar to those observed in the kidney smear (Supplementary Fig. 1c).

To initially characterize the bacteriological phenotypes of the strains, their growth sensitivity to temperature was tested (Aro et al. 2014). The optimal growth of the *Mycobacterium* strains was obtained at 25 °C in all media used, and a decreased growth rate was observed at 16 °C. However, incubation at 37 °C resulted in complete growth inhibition. Biochemical characterization using the Analytical Profile Index (API) 20E® system (BioMérieux, France), showed that all the isolates were positive for urease activity and glucose fermentation, and no other enzymatic activities were detected.

### Genomic features

PCR amplification and fragment sequencing of the 16S rRNA gene (Anahtar et al. 2016) revealed that the four isolates belonged to the *Mycobacterium* genus (Supplementary Table 2). Whole-genome sequencing results showed that the average number of reads was 15,783,247, and the average size of the genomes was 4,334,760 base pairs. The mean GC-content of the four strains was 63%, and the annotated predicted proteins were obtained from an average of 4265 coding sequences (Table 2).

**Fig. 3:**
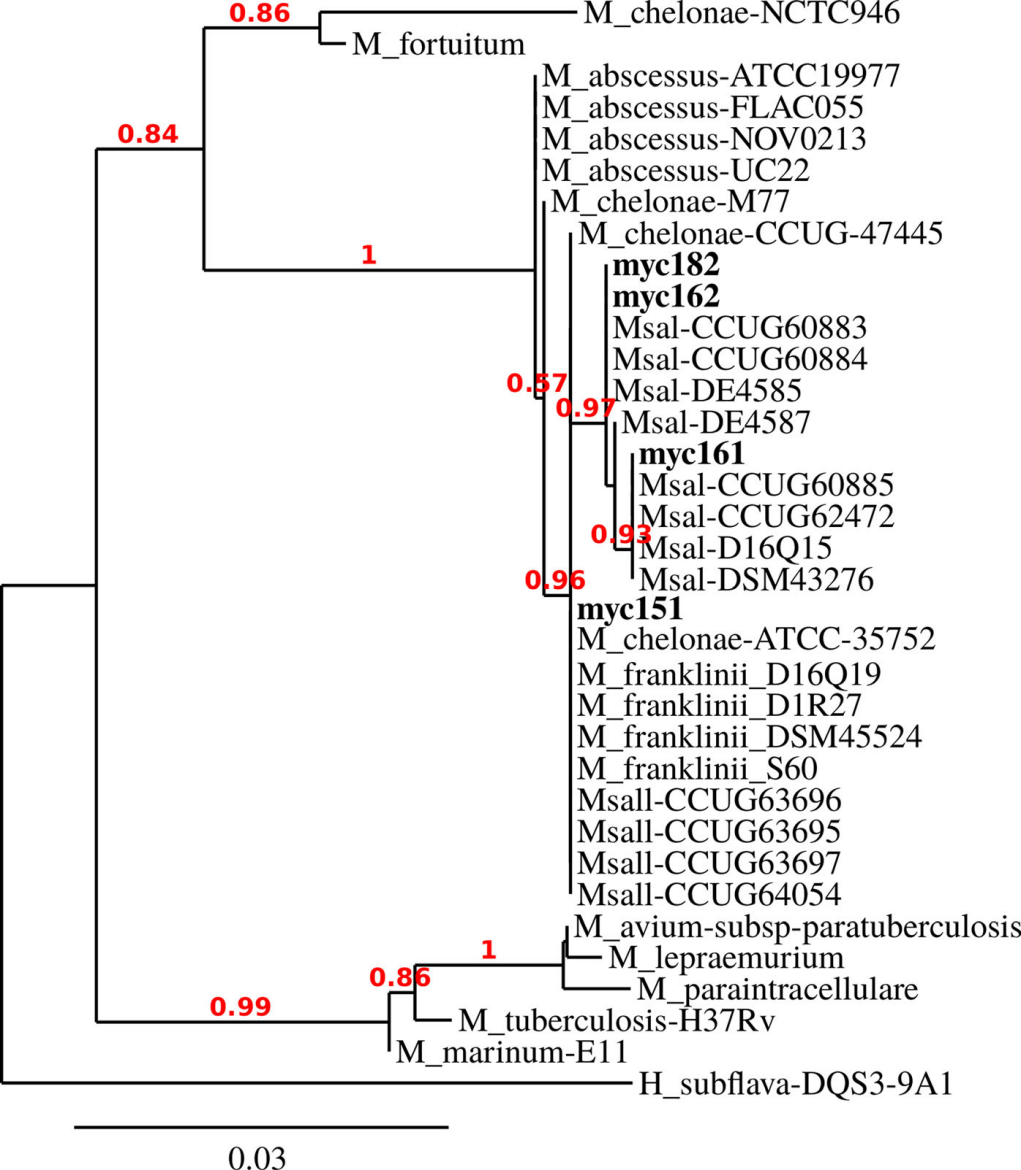
Dendrogram based on the phylogenetic relationships of the 16S rRNA gene sequences. Sequences were retrieved from the whole-genome sequencing of 4 Chilean isolates (in bold letters) and compared with 30 other *Mycobacterium* species obtained from Behra et al. (2019) and the NCBI database. The *Hoyosella subflava* DQS3-9A1 sequence was used as an outgroup, and bootstrap values are denoted in red

A BLAST search using the full-length 16S rRNA sequences retrieved from the whole-genome sequences confirmed that the bacterial strains recovered from *S. salar* (myc161, myc182, and myc162) belonged to the species *Msal* (Supplementary Table 2), whereas the strain isolated from *O. kisutch* (myc151) was identified as *Mche*. Subsequently, we used the same approximation described using the other 30 *Mycobacterium* whole-genome sequences, which were publicly available in the NCBI nucleotide database(Supplementary Table 1), to construct phylogenetic trees based on full-length 16S rRNA gene sequences using the tools provided by the R package Decipher (Wright 2016). In the phylogenetic tree constructed, the isolates myc161, myc182, and myc162 are clustered in the *Msal* branch, whereas the myc151 are clustered with the previously characterized *Msal-*like strains (Behra et al. 2019) and in close proximity to *Mfra* and some *Mche* strains. In parallel, *M. fortuitum*, *M. lepraemurium*, *M. avium*, *M. paraintracellulare*, *M. tuberculosis*, *Mma,* and two different *Mche* strains are clustered in a separate phylogenetic branch (Fig. 3). Identical results were obtained using the full-length *rpoB* sequences obtained from whole-genome annotations (Supplementary Fig. 2).

### Average Nucleotide Identity (ANI)

**Fig. 4:**
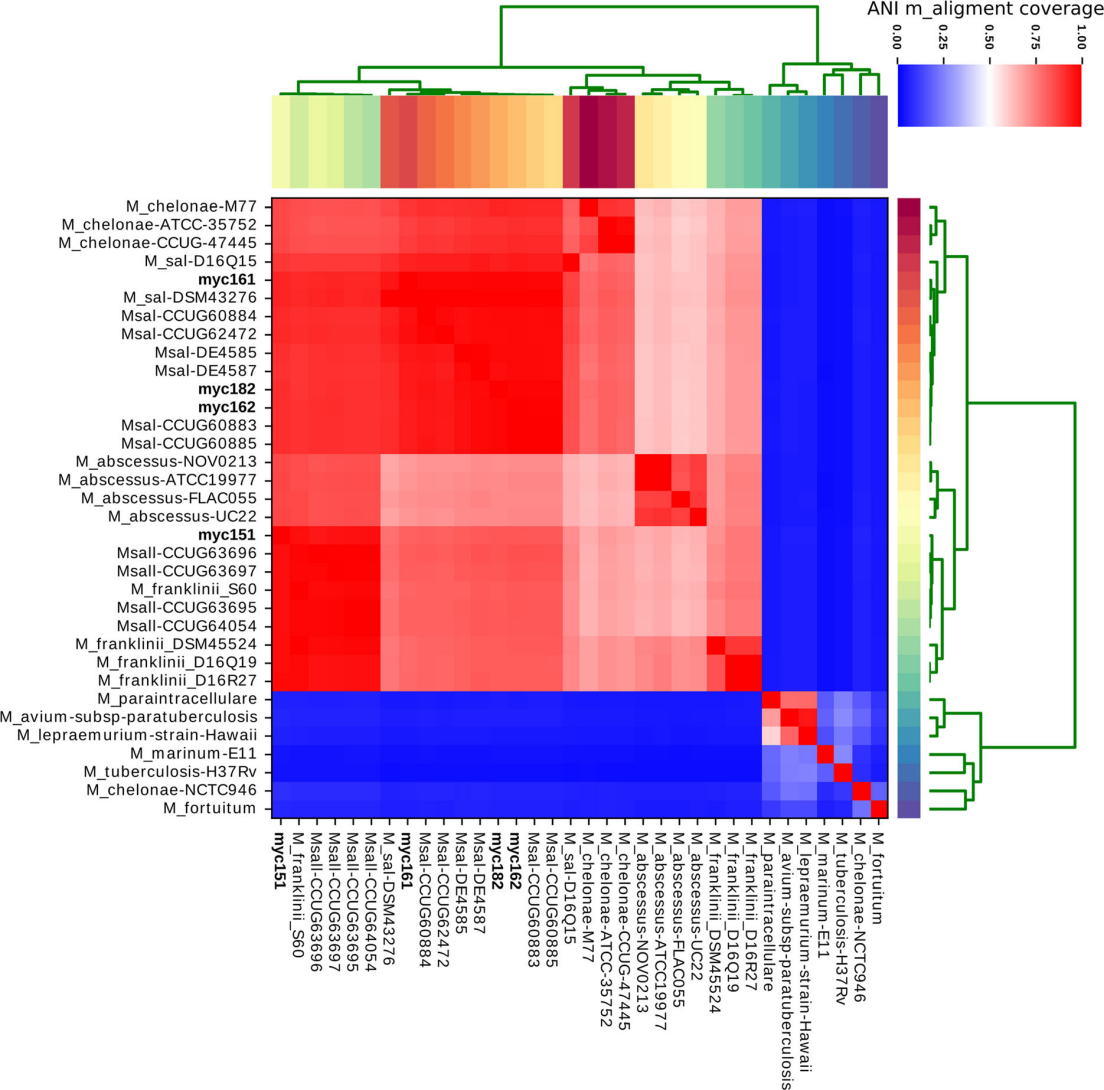
Average Nucleotide Identity (ANI) based on the whole-genome sequences of 34 genomes belonging to the *Mycobacterium* genus*.* Clustering of the whole genomes was based on the MUMmer alignment of the input sequences and is represented by the heatmap. The ANI m_aligment coverage represents the percentage of nucleotide identity in the matching regions among genomes (coloured blue for low coverage and red colour for high alignment coverage). The horizontal tree depicts the clustering of column-wise dendrogram

To understand the interrelationship between the four *Mycobacterium* strains and other *Mycobacterium* species, we performed unsupervised clustering aided by ANI analysis (Konstantinidis and Tiedje 2005) using a total of 34 *Mycobacterium* whole-genome sequences, including 30 previously published genomes (Supplementary Table 1). First, the four isolates were clustered with genomes belonging to the *Msal*, *Msal-*like, *Mfra*, *Mabs*, and *Mche* species, yielding ANI scores > 50% and forming five related clusters. Seven genomes belonging to *M. fortuitum*, *Mche*, *M. tuberculosis*, *Mma*, *M. lepraemurium*, *M. avium,* and *M. paraintracellulare* clustered separately with ANI scores < 25% (Fig. 4). Second, ANI alignment coverage revealed that myc162, myc182, and myc161 were clustered together with the *Msal* genomes. However, myc151 was clustered with the genomes of the *Msal*-like strains and those of some *Mfra* strains, in accordance with the results obtained using the full-length 16S rRNA and *rpoB* sequences (Fig. 3 and Supplementary Fig. 2), thereby indicating that this strain does not correspond to *Msal*.

Finally, according to the 95% identity threshold (Pritchard et al. 2016), myc162, myc182, and myc161 were most similar in identity to the *Msal* genomes, whereas myc151 potentially belongs to the *Mfra* and *Msal-*like species, a genetically separate phylogenetic branch (Behra et al. 2019).

### Comparative analysis of core- and pan-genomes

The annotated assemblies obtained by the Prokka tool (Seemann 2014) from the 27 whole-genomes belonging to the *Msal*, *Msal-like*, *Mfra*, *Mabs*, and *Mche* species, were then used to calculate the core-genome and pan-genome, in which gene presence for each genome was represented in parallel to compare similarities between species (Supplementary Fig. 3). Overall, annotated gene number ranged from 4,071 to 5,396, with myc151 and *Mfra*-DSM45524 containing the least and the greatest number of genes, respectively. Genome analysis of the *Mycobacterium* species yielded 23,935 predicted genes, of which 861 genes were shared across all species and comprised the core genome.. Furthermore, after comparing the gene content patterns of the isolates, we found that myc182 and myc162 displayed the greatest core similarities to *Msal* strains CCUG60883, CCUG60885, DE4585, and DE4587, whereas myc161 shared core patterns with the genomes of *Msal* CCUG60884, CCUG62472, and DSM43276 (Supplementary Fig. 4). Lastly, myc151 shared a core pattern with *Mfra* and the *Msal-*like species, supporting the results obtained via the 16S rRNA phylogeny and ANI analysis (Figs. 2, 4 and Supplementary Fig. 4).

### Functional classification of annotated genes

**Fig. 5:**
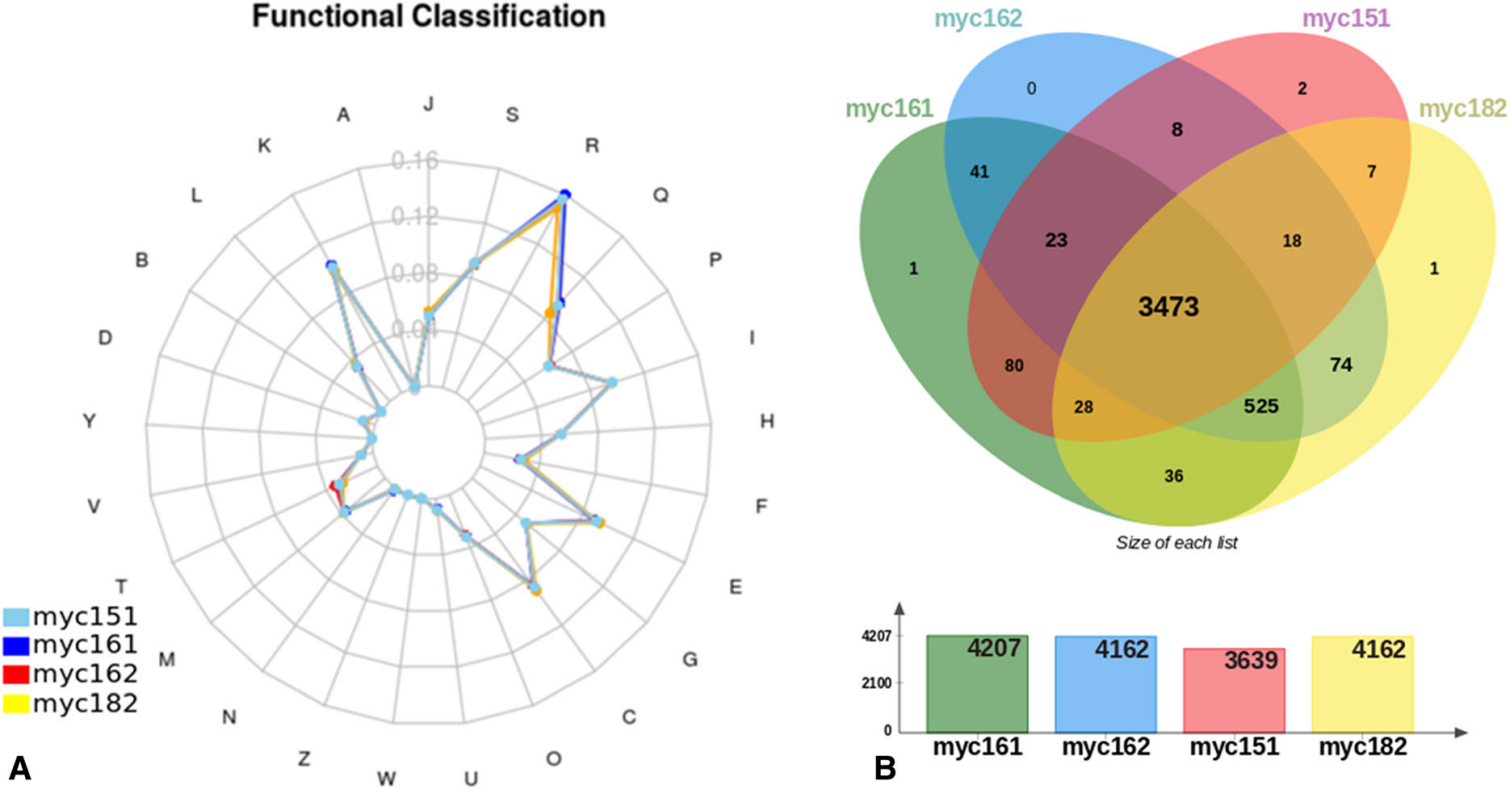
Functional classification of annotated genes of *Mycobacterium* isolates from Chile. (**a**) Spider web diagram of the abundance profile of Clusters of Orthologous Groups (COGs) identified from whole-genome sequencing. (**b**) Venn diagram of the comparative analysis between Chilean isolates showing the common and unique annotated predicted genes

A protein function clustering of the 4 Chilean isolates utilizing the COG database (Tatusov 2000) was performed to describe the functional patterns of annotated genes. All four isolates displayed almost identical functional categories, being the “R: general functional prediction only” and “S: function unknown” (Fig. 5a) the most enriched COG categories. The other functional COG categories included transcription, secondary metabolites biosynthesis, transport, and catabolism (categories K and Q). Subsequently, a differential analysis of orthologous gene clusters between isolates was conducted, and we found that the four strains formed 4,317 clusters of orthologs, including 3,473 core gene orthologs and 4 unique elements without orthologs (Fig. 5b). Interestingly, those belonging specifically to myc151, myc161 and myc182, were already described for some *Mycobaterium* isolates. The unique element for myc161 was predicted to be a hypothetical protein, that had been previously predicted in *Mche* (NCBI Acc. Number: WP_200996060). For myc151, one element matched with a PE family protein annotated in *Msal* (WP_134081245) and *Mfra* (WP_134048526), and the second element was predicted to be a hypothetical protein, annotated in *Mabs* (NCBI Acc. Number: SKT85326). The unique element belonging to myc182 was predicted to be a family type VII secretion target that was previously identified in *Mfra* (WP_070937634) and *Msal* (WP_134062353). Altogether, these observations suggest that the unique elements identified are not strain specific, and instead belong more broadly to the *Mycobacterium* clade.

### Identification of virulence and antimicrobial-resistance genes

Next, we surveyed the whole-genome of the four isolates for the presence of genes encoding virulence factors (VF) and antibiotic resistance (AR). The isolates shared the presence of several VF genes, including *ideR, icl*, *mbtH*, *relA*, and *phoP*. Interestingly, *mbtH* encodes the siderophore protein mycobactin (VF0299), which is involved in the shuttling of free extracellular iron ions into the cytoplasm of mycobacterial cells (Supplementary Fig. 5). Besides, *ideR* also regulates functions related to iron metabolism, suppressing genes that encode proteins involved in siderophore synthesis (mbtA-G), secretion (mmpL4/5, mmpS4/5) and uptake (irtAB) (Zondervan et al. 2018).

Regarding AR genes, only the strains myc162 and myc182 encoded genes associated with the resistance to carbapenem, such as CRP-1 and carbapenem-hydrolysing class A beta-lactamase. This result was consistent with the high amount of genetic similarity of myc162, myc182, and some *Msal* strains, specifically *Msal* CCUG60883, CCUG60885, DE4585, and DE4587, which also exhibit resistance to carbapenem. Finally, we showed that the other analysed *Mycobacterium* strains possessed genes for resistance to beta-lactam, tetracycline, gentamycin, macrolide, and rifampin (Supplementary Table 3).

## Discussion

This study comprehensively analysed the genomes of serveral *Mycobacterium* strains. The presence of this genus had been previously reported years ago in Chile (Aro et al. 2014). Here, we expand a previous the report of mycobacteriosis in Chile, characterising the whole-genome of 4 isolates derived from salmon freshwater aquaculture facilities. This new description includes whole-genome analyses of four isolated bacterial strains, three of which (myc161, myc182 and myc162) were from *S. salar,* and one of which (myc151) was from *O. kisutch*. All four were isolated from freshwater systems, which suggests that these *Mycobacterium* strains specifically affect the freshwater stage of salmonid farming. First, macroscopically, the fish displayed biological characteristics consistent with previous descriptions of infections caused by members of the *Mycobacterium* genus(Aro et al. 2014; Bruno et al. 1998; Brocklebank et al. 2003; Keller et al. 2018; Luo et al. 2018; Parikka et al. 2012). Second, our bacteriological findings and 16S rRNA PCR results confirmed that the isolates belong to the *Mycobacterium* clade. Whole-genome sequencing confirmed that three of the isolates: myc161, myc182, and myc162, belonged to the *Msal* species, while the isolate myc151 was closely related to the new *Mfra/Msal*-like phylogenetic branch previously described by Behra et al. (2019), wherein the authors identified significant genomic differences between the strains. As such, these *Msal*-like strains belong to a single species according to their ANI score.

In accordance with previously reported data, we observed that the *Mfra* strains displayed patterns that were similar, but not identical, to those of the *Msal*-like strains in terms of their 16S rRNA, whole-genome, and core- and pangenome sequences as the *Msal*-like strains. This supports the existence of an independent *Mfra/Msal*-like species closely related to *Mfra*. Interestingly, only the strain isolated from coho salmon was identified as belonging to the *Mfra*/*Msal*-like species, whereas those from the Atlantic salmon were identified as *Msal* (Figs. 3, 4 and Supplementary Fig. 2).

The bacterium *Mfra* was first classified as a member of the *Mche-Mabs* complex (MCAC) by Simmon et al. (2011), who isolated it from patients with underlying lung conditions. Although the exact source of the disease-causing pathogen is unknown, another study (Van Ingen et al. 2010) has detected *Mfra* in municipal water sources, suggesting an environmental origin that may be region-specific. Collectively, these studies suggest that terrestrial *Mfra*/*Msal*-like infections may directly originate from nearby water sources. Hence, this is the first study to report a newly proposed species, which potentially cause mycobacteriosis in salmonids, belonging to the *Mfra*/*Msal*-like cluster from a Chilean freshwater breeding system, highlighting the need to monitor these mycobacteriosis-related diseases in aquaculture settings. On the other hand, *Msal* has been previously reported to cause mycobacteriosis outbreaks in *S. salar* and other fish species (Whipps et al. 2007; Zerihun et al. 2011b, a; Austin and Austin 2016). However, no evidence has thus far suggested that *Msal* causes infections in terrestrial animals. Interestingly, the mycobacteriosis described here manifested differently in coho and Atlantic salmon species. Mycobacteriosis in *S. salar* was characterized by internal granulomas, whereas *O. kisutch* mainly exhibited chronic peritonitis (Fig. 1). Nevertheless, we cannot eliminate the possibility that different *Mycobacterium* species cause distinct disease manifestations.

Recent advances in sequencing technology facilitated the identification of several *Mycobacterium* species and subspecies based on phylogenetic algorithms from multiple genomic databases, thus generating more accurate and robust phylogenetic classification. One such example is Das et al. (2018), who analysed the genome sequences of 19 *Mma* strains using comparative genomics, ANI analysis, and phylogenetic trees to cluster the strains into two distinct branches. Similarly, Behra et al*.* (2019) performed several comparative genomic analyses and concluded that *Msal*-like isolates and *Mfra* isolates are similar enough to constitute a single species. Recently, a study performed on isolates recovered from gilthead seabream identified *Mma* as an etiological agent of mycobacteriosis using molecular and mass spectrometry (MALDI-TOF) analyses (Davidovich et al. 2020). In this work, in addition to 16S rRNA analysis for phylogenetical classification, we utilized multiple genomic tools to assign the identities and characterise the strains isolated. Altogether, this work highlights the importance of reviewing previous classifications of infectious *Mycobacterium* species using novel scientific tools and several bioinformatic approaches.

One limitation of our study was the low number of Chilean isolates analysed. However, the identification of new bacterial isolates from aquaculture facilities may confirm the presence of the *Msal-like* strains. Our findings pave the way for the further investigation and monitoring of this bacterial genus as a potential etiological agent of an emerging disease, namely mycobacteriosis in aquaculture systems. Interestingly, virulence factors were found in all the strains studied that have been reported in other mycobacterium spp.; *phoP* (Ryndak et al. 2008), *mbtH* (Baltz 2011), ideR (Zondervan et al. 2018), among others. These last two genes, regulate iron homeostasis through various mechanisms, and play a crucial role in mycobacterium tuberculosis virulence (Pandey and Rodriguez 2014). According to, this VFs has been used as an important target to guide the search for new drugs (Wang et al. 2016; Rohilla et al. 2017; Salimizand et al. 2017). If strategies are found that successfully inhibit these targets, they may be used to treat multiple other mycobacteriosis of human and animal origin. In addition, the presence of AR genes in myc162 and myc182 that encode genes for resistance to carbapenem, points to the need to study the minimum inhibitory antibiotic concentrations for these and other new isolates. Finally, our findings will serve as guide to future studies aiming to provide insights into mycobacterial biology and pathogenicity using a combination of several tools simultaneously.

## Declarations

## Supplementary information

Below is the link to the electronic supplementary material.

Supplementary file1 (DOCX 4.90 mb)

## Data Availability

The whole-genome datasets generated during the current study are available in the ENA-EMBL (https://www.ebi.ac.uk/ena/browser/view/PRJEB38737), and their accession numbers are provided in Table 2 and Supplementary Table 1. All other data generated or analyzed during this study are included in this published article and its supplementary information files.
