# Correction to: Whole-Genome sequencing and comparative genomics of *Mycobacterium* spp. from farmed Atlantic and coho salmon in Chile

**DOI:** 10.1007/s10482-021-01606-7

**Published:** 2021-07-09

**Authors:** Rudy Suarez, Karina Kusch, Claudio D. Miranda, Tianlu Li, Javier Campanini, Phani Rama Krishna Behra, Luis Aro, Alexis Martínez, Marcos Godoy, Daniel A. Medina

**Affiliations:** 1 Centro de Investigaciones Biológicas Aplicadas, Diego de Almagro Norte 1013, Puerto Montt, Chile; 2 Programa de Magíster en Acuicultura, Facultad de Ciencias del Mar, Universidad Católica del Norte, Larrondo 1281, Coquimbo, Chile; 3 Laboratorio de Patobiología Acuática, Departamento de Acuicultura, Universidad Católica del Norte, Larrondo 1281, Coquimbo, Chile; 4 Epigenetics and Immune Disease Group, Josep Carreras Leukaemia Research Institute, Badalona, Barcelona, Spain; 5 Facultad de Medicina y Ciencia, Universidad San Sebastián, Lago Panguipulli 1390, Puerto Montt, Chile; 6 Department of Cell and Molecular Biology, Biomedical Centre, Box 596, 751 24 Uppsala, Sweden; 7 Benchmark Genetics, Santa Rosa 560 oficina 25 B, Puerto Varas, Chile; 8 Cardonal S/N, AquaChile, Puerto Montt, Chile; 9 Programa Cooperativo Doctorado en Acuicultura, Universidad Católica del Norte, Larrondo 1281, Coquimbo, Chile; 10 Laboratorio de Biotecnología Aplicada, Facultad de Medicina Veterinaria, Universidad San Sebastián, Lago Panguipulli 1390, Puerto Montt, Chile

## Correction to: Antonie van Leeuwenhoek 10.1007/s10482-021-01592-w

In the original publication of the article, the affiliation of the author “Marcos Godoy” was not correctly tagged. The author is affiliated with “Centro de Investigaciones Biológicas Aplicadas, Diego de Almagro Norte 1013, Puerto Montt, Chile”, “Programa Cooperativo Doctorado en Acuicultura, Universidad Católica del Norte, Larrondo 1281, Coquimbo, Chile” and “Laboratorio de Biotecnología Aplicada, Facultad de Medicina Veterinaria, Universidad San Sebastián, Lago Panguipulli 1390, Puerto Montt, Chile”.

The original article has been corrected.

