# Supplementary material for: Whole-Genome sequencing and comparative genomics of Mycobacterium spp. from farmed Atlantic and coho salmon in Chile
Source: Antonie Van Leeuwenhoek. 2021 May 30;114(9):1323–36. doi: 10.1007/s10482-021-01592-w (PMC8379129; doi:10.1007/s10482-021-01592-w)
Supplement: Supplementary file 1 — Supplementary file1 (DOCX 4.90 mb) [file 10482_2021_1592_MOESM1_ESM.docx]

**Supplementary Fig. 1** **Microbial visualization of *Mycobacterium* isolates.** (a) Plate growth in MAOA medium after 5 days of culture at 25 °C. (b) Ziehl–Neelsen staining. (c) Direct microscopy observation of kidney smears stained with Ziehl–Neelsen


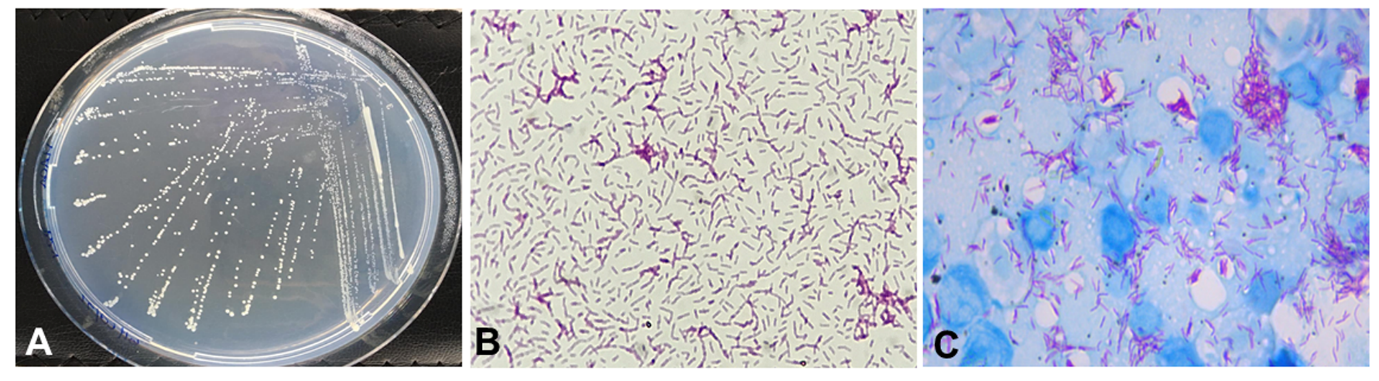


**Supplementary Fig. 2** Dendrogram based on the phylogenetic analysis using all PROKKA annotated *rpoB1* sequences. The *Hoyosella subflava* DQS3-9A1 sequence was used as an outgroup, and bootstrap values are denoted in red


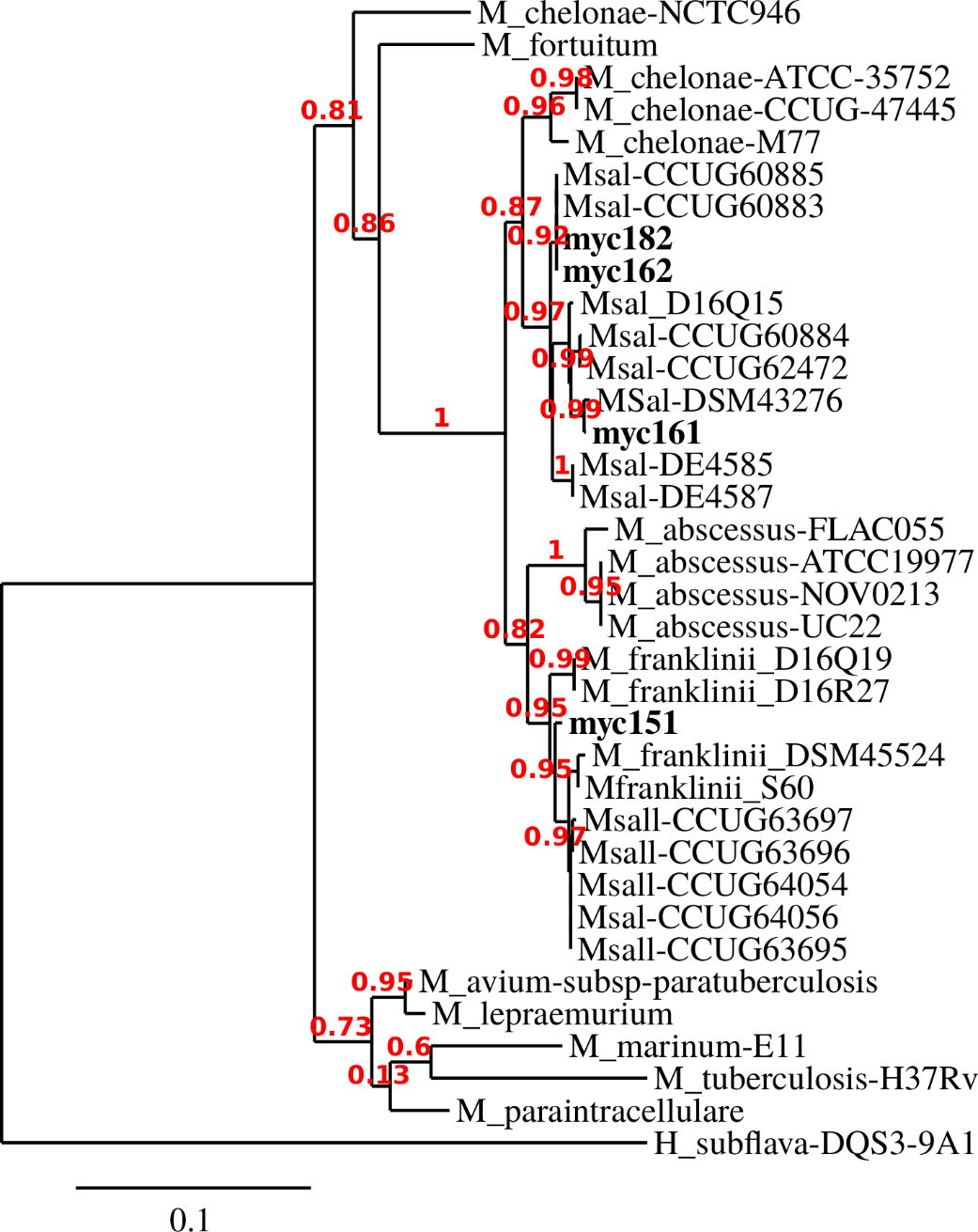


**Supplementary Fig. 3** **Comparative overview of *Mycobacterium* pan and core genomes**. Grey regions denote the common and unique annotated genes predicted, showing from top to bottom the core-genome shared between strains and the entire pangenome (the sum of all core-genomes). The number of annotated genes is indicated in the right of each genome


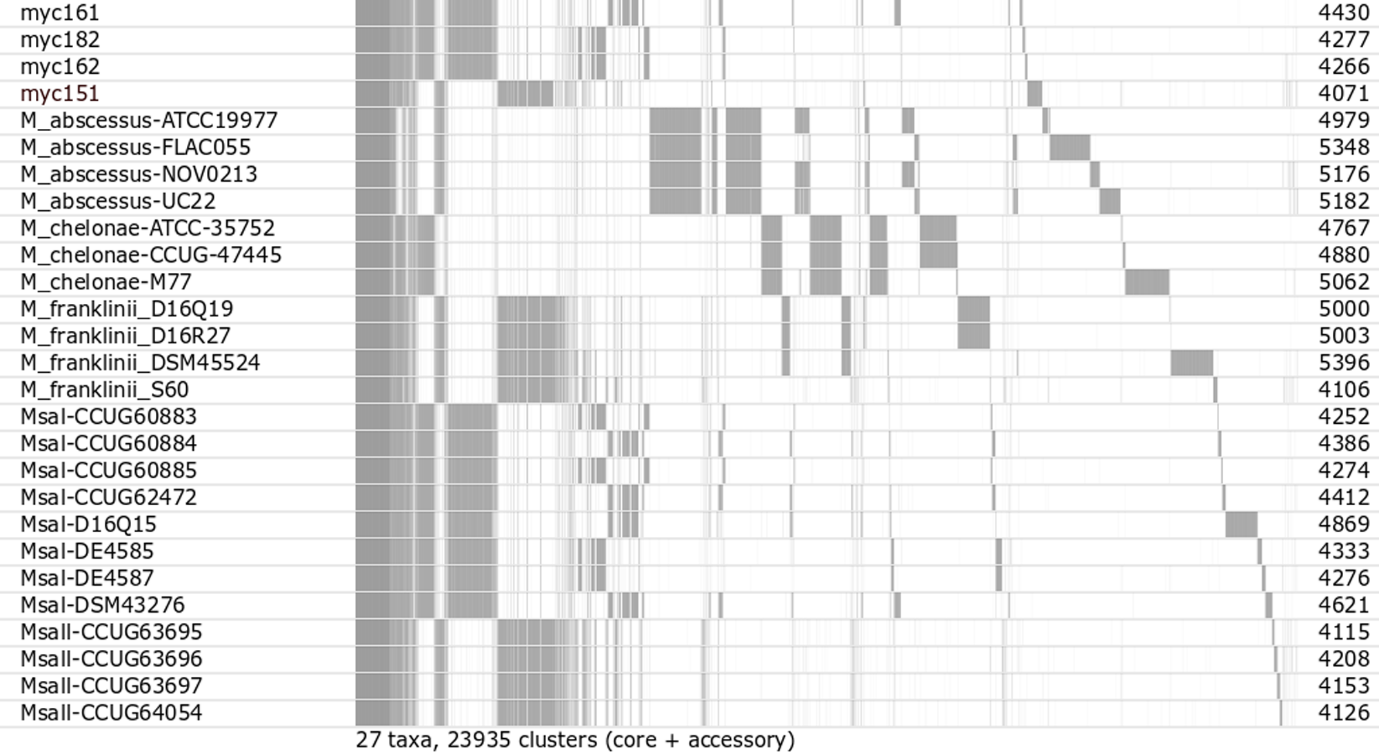


**Supplementary Fig. 4** **Pan-genome of *Mycobacterium* spp. grouped with the *Mycobacterium* species with best core match.** The annotated genomes were clustered using the best common coverage between similar strains, showing their extended core genomes and unique predicted genes


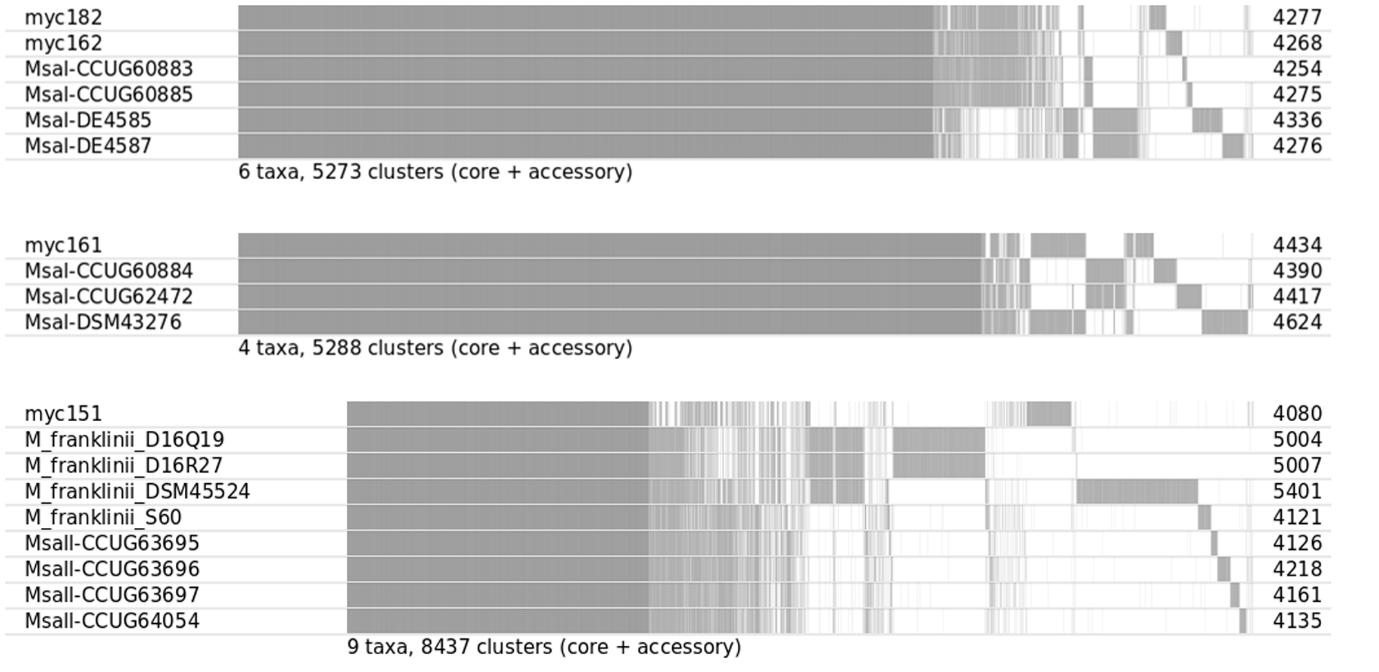


**Supplementary Fig. 5 Heatmap of the distribution of virulence genes identified based on the virulence factor database (VFDB).** The colours represent the numbers of repetitions of the virulence genes in each genome analysed. The dendrogram was drawn based on the hierarchical clustering of the Euclidean distance between values.


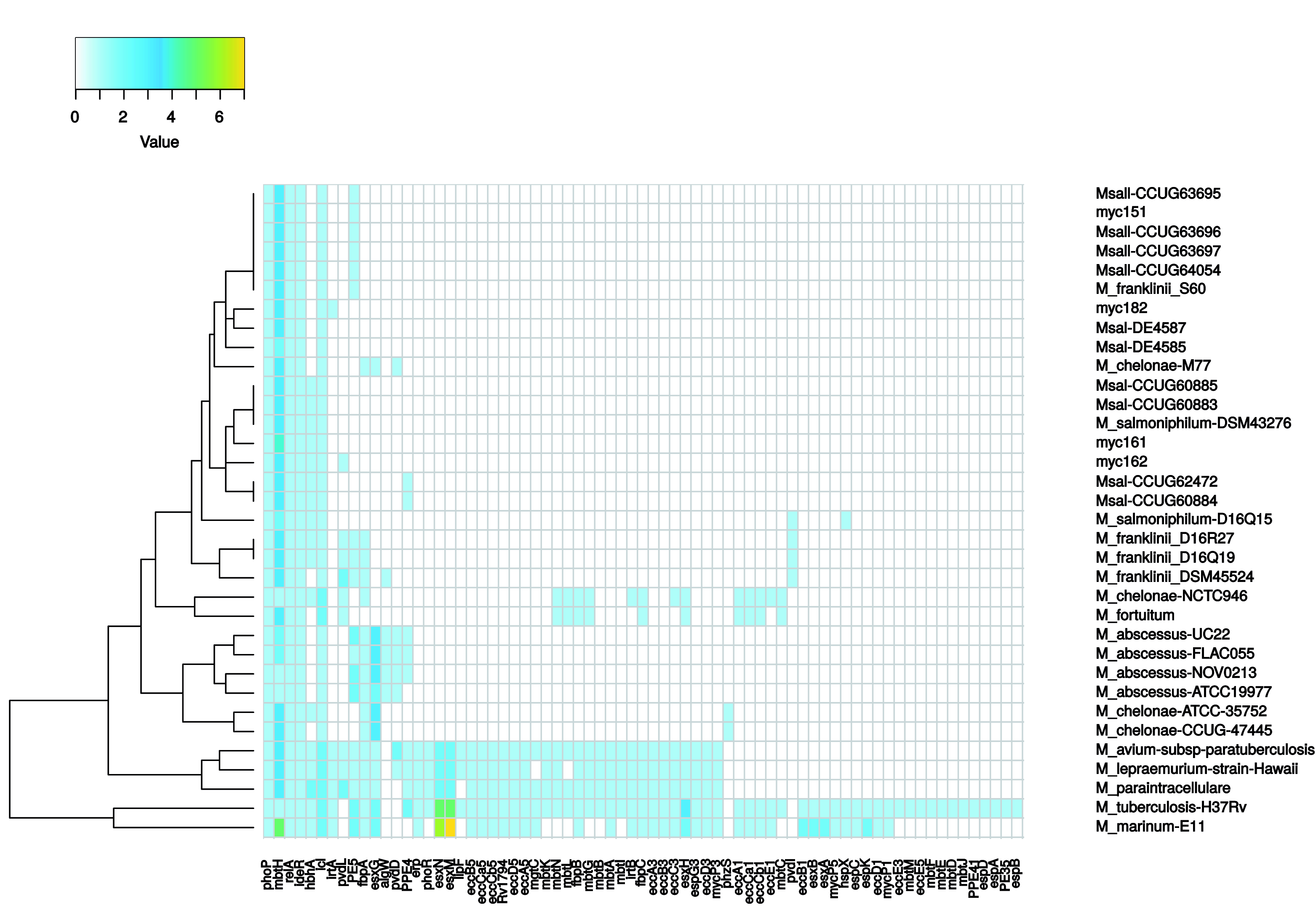


**Supplementary Table 1**. Accession numbers and sources of whole genome sequences used in this study.

| M_lepraemurium | NZ_CP021238 |  |  |  |  |
| --- | --- | --- | --- | --- | --- |
| M_marinum-E11 | NZ_HG917972 |  |  |  |  |
| M_paraintracellulare | NC_016948 |  |  |  |  |
| Msal-CCUG60883 | NZ_PECM00000000 |  |  |  |  |
| Msal-CCUG60884 | NZ_PECL01000000 |  |  |  |  |
| Msal-CCUG60885 | NZ_PECK01000000 |  |  |  |  |
| Msal-CCUG62472 | NZ_PECJ00000000 |  |  |  |  |
| Msal-DE4585 | NZ_PECH01000000 |  |  |  |  |
| Msal-DE4587 | NZ_PECI01000000 |  |  |  |  |
| Msall-CCUG63695 | NZ_PECE01000000 |  |  |  |  |
| Msall-CCUG63696 | NZ_PECD01000000 |  |  |  |  |
| Msall-CCUG63697 | NZ_PECC01000000 |  |  |  |  |
| Msall-CCUG64054 | NZ_PECB01000000 |  |  |  |  |
| Msal-D16Q15 | NZ_MAFE01000000 |  |  |  |  |
| Msal-DSM43276 | NZ_CP024633 |  |  |  |  |
| M_tuberculosis-H37Rv | NC_000962 |  |  |  |  |
| H_subflava-DQS3-9A1* | NC_015564 |  |  |  |  |
|  |  |  |  |  |  |
| *This genome was used as outgroup for phylogenetical representation of 16S rRNA and rpob gene sequence. | | | | | |

**Supplementary Table 2**. Molecular identification of each bacterial strain.

| Strain | 16S PCR BLAST identification | ldentity (%) | NCBI Accesion match | No. of base pairs | Entire 16S & 23S NGS sequence BLAST identification | ldentity (%) | NCBI Accesion match | No. 16S rRNA bp |
| --- | --- | --- | --- | --- | --- | --- | --- | --- |
| myc161 | *Mycobacterium salmoniphilum* | 96.6 % | CP024633 | 804 | *Mycobacterium salmoniphilum* | 100 % | CP024633 | 1436 |
| myc182 | *Mycobacterium salmoniphilum* | 97.5 % | KC333054 | 814 | *Mycobacterium salmoniphilum* | 99.79 % | CP024633 | 1436 |
| myc162 | *Mycobacterium salmoniphilum* | 100 % | CP024633 | 812 | *Mycobacterium salmoniphilum* | 99.79 % | CP024633 | 1436 |
| myc151 | *Mycobacterium chelonae* | 100 % | CP034383 | 802 | *Mycobacterium chelonae* | 100 % | CP034383 | 1436 |
|  |  |  |  |  |  |  |  |  |
| ^a^16S rRNA sequences were retrieved from whole-genomic sequences and were searched in the NCI-BLAST database. | | | | | |  |  |  |

| Supplementary Table 3. Identification of annotated antimicrobial-resistance genes using the Resfinder and NCBI AMRFinderPlus databases | | | |
| --- | --- | --- | --- |
|  |  |  |  |
|  |  |  |  |
|  |  |  |  |
| **Gene identified** | **Product** | **Resistance** | ***Mycobacterium* genome** |
| *blaCRP-1* | carbapenem-hydrolyzing class A beta-lactamase CRP-1 | Carbapenem | myc182, myc162, Msal-CCUG60883, Msal-CCUG60885 |
| *penI_Bp* | PenI family class A extended-spectrum beta-lactamase | Beta-Lactam | *M. abscessus-FLAC055, M. franklinii_DSM45524* |
| *blaALG6-1* | subclass B3 metallo-beta-lactamase ALG6-1 | Carbapenem | *M. chelonae-ATCC-35752, M. chelonae-CCUG-47445* |
| *erm(38)* | 23S rRNA (adenine(2058)-N(6))-methyltransferase Erm(38) | Macrolide | *M. chelonae-NCTC946, M. tuberculosis-H37Rv* |
| *blaS* | class A beta-lactamase | Beta-Lactam | *M. chelonae-NCTC946* |
| *tet(V)* | tetracycline efflux MFS transporter Tet(V) | Tetracycline | *M. chelonae-NCTC946, M. fortuitum* |
| *aac(2')-Ib* | aminoglycoside N-acetyltransferase AAC(2')-Ib | Gentamicin/Tobramcyin | *M. chelonae-NCTC946, M. fortuitum* |
| *erm(39)* | 23S rRNA (adenine(2058)-N(6))-methyltransferase Erm(39) | Macrolide | *M. fortuitum* |
| *blaF* | class A beta-lactamase | Beta-Lactam | *M. fortuitum* |
| *rox* | rifampin monooxygenase Rox | Rifampin | *M. fortuitum, M. paraintracellulare* |
| *arr-Ms* | NAD(+)--rifampin ADP-ribosyltransferase | Rifamycin | *M. fortuitum, M. marinum-E11* |
| *aph(3'')-Ic* | aminoglycoside O-phosphotransferase APH(3'')-Ic | Streptomycin | *M. fortuitum* |
| *aac(2')-Ic* | aminoglycoside N-acetyltransferase AAC(2')-Ic | Gentamicin/Tobramcyin | *M. marinum-E11* |
| *iri* | rifampin monooxygenase Iri | Rifampin | *M. marinum-E11* |
| *blaCRH-1* | carbapenem-hydrolyzing class A beta-lactamase CRH-1 | Carbapenem | *Msal-CCUG60883, Msal-CCUG60885, Msal-DE4585, Msal-DE4587* |
| *erm(37)* | 23S rRNA (adenine(2058)-N(6))-methyltransferase Erm(37) | Macrolide | *M. tuberculosis-H37Rv* |
| *blaA_Mtub* | class A beta-lactamase BlaA | Beta-Lactam | *M. tuberculosis-H37Rv* |
